# The complete mitogenome of *Metopograpsus quadridentatus* and phylogenetic analysis

**DOI:** 10.1080/23802359.2018.1524272

**Published:** 2018-10-27

**Authors:** Zhengfei Wang, Chenyao Ji, Ziqian Wang, Dan Tang, Qiong Wu, Huayun Guo, Yitao Tao

**Affiliations:** Jiangsu Key Laboratory for Bioresources of Saline Soils, Jiangsu Synthetic Innovation Center for Coastal Bio-agriculture, Jiangsu Provincial Key Laboratory of Coastal Wetland Bioresources and Environmental Protection, School of Ocean and Biological Engineering, Yancheng Teachers University, Yancheng, China

**Keywords:** Mitogenome, Brachyura, Grapsidae, *Metopograpsus quadridentatus*, phylogentic

## Abstract

The complete mitochondrial genome of *Metopograpsus quadridentatus* was determined to be 15,520 bp in length. It consists of 13 protein-coding genes, 22 transfer RNAs, 2 ribosomal RNAs and a control region. There are 13 overlapping regions in the genome with 1 to 25 bp length. The largest overlapping region is located between *nad1* and *trnL1*. The AT-skew and GC-skew for the whole mitogenome are both negative, indicating a higher occurrence of Ts than As and Cs than Gs. The molecular data here we presented could play a useful role to study the evolutionary relationships and population genetics of Grapsidae crabs.

*Metopograpsus quadridentatus*, belongs to the genus *Metopograpsus*, mainly lives in mangroves and intertidal areas of the eastern Indian and West Pacific oceans, from Singapore to southern China (Ng et al. 2008). The genus *Metopograpsus* (H. Milne Edwards, 1853) belongs to the family Grapsidae (within  Crustacea: Decapoda: Thoracotremata : Brachyura) and includes six species of intertidal crabs from sheltered rocky shores or mangroves and associated muddy areas (Paulay 2007). The taxonomy of the genus *Metopograpsus* has been questioned due to minor diagnostic morphological differences among species (Fratini et al. 2018). Therefore, to facilitate the future researches of taxonomic resolution, population genetic structure and phylogeography, the complete mitochondrial genome of *M. quadridentatus* was determined.

The samples were collected from the coastal intertidal zone of Shanghai (China) on 19 September 2017 (121°54'38″E, 30°51'28″N). All samples were stored at the Jiangsu Provincial Key Laboratory of Coastal Wetland Bioresources and Environmental Protection, Yancheng Teachers University, Yancheng, Jiangsu Province, China. Total DNA was extracted from the muscle tissue and using the Aidlab Genomic DNA Extraction Kit (Aidlab Biotech, Beijing, China). The mitogenomes of *M. quadridentatus* were sequenced by next-generation sequencing (Illumina HisSeq 4000), and clean data without sequencing adapters were *de novo* assembled by the NOVOPlasty software (Dierckxsens et al. 2017). The mitogenome of *M. quadridentatus* is a closed circular molecule 15,520 bp in size. The gene content is typical of Decapoda mitochondrial genomes, including 13 PCGs (*cox1*–*3*, *nad1*–*6*, *nad4L*, *cob*, *atp6* and *atp8*), 2 rRNA genes (*rrnS* and *rrnL*), 22 tRNA genes and a major non-coding region known as the CR. Twenty-three genes are encoded on the heavy (+) strand while the remaining 14 genes (4 of the 13 PCGs, 8 tRNAs and 2 rRNAs) are located on the light (−) strand. There are 13 overlapping regions in the genome with 1 to 25 bp length. The genome shows 19 intergenic sequences varying from 1 to 80 bp in size. The AT-skew and GC-skew for the whole mitogenome are both negative, indicating a higher occurrence of Ts than As and Cs than Gs. The mitogenome of *M. quadridentatus* has been deposited in GenBank under accession number MH310445.

To reconstruct the phylogenetic relationship among crabs, the complete mitogenomes of other 61 Brachyura and one outgroup (*Clibanarius infraspinatus*) were obtained from the GenBank database (https://www.ncbi.nlm.nih.gov/genbank/). The concatenated set of nucleotide sequences were used for phylogenetic analysis, which was performed with the BI and ML methods using MrBayes v3.2.6 (Huelsenbeck and Ronquist 2001) and RaxML (Stamatakis 2014), respectively. In this study, both the BI and ML analyses showed that each family in the tree formed a monophyletic clade (Figure 1). From the phylogenetic tree, we found *M. quadridentatus* and (*Grapsus tenuicrustatus* + *Pachygrapsus crassipes*) are clustered in one branch with high nodal support value (BI posterior probabilities [PP] = 100, Figure 1). Additionally, the relationship among Sesarmidae, Xenograpsidae, Grapsidae, Varunidae, Potamidae, Bythograeidae, and Portunidae is also identified and is largely consistent with previous researches (Shen et al. 2013; Wang et al. 2018a,b).

**Figure 1.**
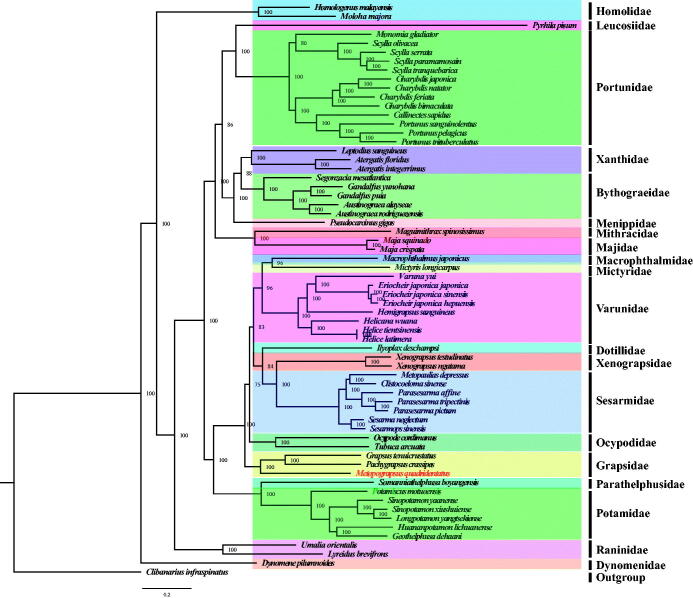
Phylogeny of Brachyura based on nucleotide sequences. The phylogenetic tree was inferred from the nucleotide sequences of 13 mitogenome PCGs using BI and ML methods. Numbers on branches indicate posterior probability (BI). *Clibanarius infraspinatus* was used as outgroup. The genbank accession numbers for all of the sequences is listed as follows: *Atergatis floridus* NC_037201.1, *Atergatis integerrimus* NC_037172.1, *Austinograea alayseae* NC_020314.1, *Austinograea rodriguezensis* NC_020312.1, *Callinectes sapidus* NC_006281.1, *Charybdis feriata* NC_024632.1, *Charybdis japonica* NC_013246.1, *Charybdis natator* NC_036132.1, *Clibanarius infraspinatus* NC_025776.1, *Clistocoeloma sinense* NC_033866.1, *Dynomene pilumnoides* KT182070.1, *Eriocheir japonica hepuensis* NC_011598.1, *Eriocheir japonica japonica* NC_011597.1, *Eriocheir japonica sinensis* NC_006992.1, *Gandalfus puia* NC_027414.1, *Gandalfus yunohana* NC_013713.1, *Geothelphusa dehaani* NC_007379.1, *Grapsus tenuicrustatus* NC_029724.1, *Charybdis bimaculata* MG489891.1, *Helicana wuana* NC_034995.1, *Helice latimera* NC_033865.1, *Helice tientsinensis* NC_030197.1, *Hemigrapsus sanguineus* NC_035307.1, *Homologenus malayensis* NC_026080.1, *Huananpotamon lichuanense* NC_031406.1, *Ilyoplax deschampsi* NC_020040.1, *Leptodius sanguineus* NC_029726.1, *Longpotamon yangtsekiense* NC_036946.1, *Lyreidus brevifrons* NC_026721.1, *Macrophthalmus japonicus* NC_030048.1, *Maguimithrax spinosissimus* NC_025518.1, *Maja crispata* NC_035424.1, *Maja squi-nado* NC_035425.1, *Metopaulias depressus* NC_030535.1, *Mictyris longicarpus* NC_025325.1, *Moloha majora* NC_029361.1, *Monomia gladiator* NC_037173.1, *Ocypode cordimanus* NC_029725.1, *Potamiscus motuoensis* KY285013.1, *Pachygrapsus crassipes* NC_021754.1, *Parasesarma affine* MH310444, *Parasesarma pictum* MG580780, *Parasesarma tripectinis* NC_030046.2, *Portunus pelagicus* NC_026209.1, *Portunus sanguinolentus*NC_028225.1, *Portunus trituberculatus* NC_005037.1, *Pseudocarcinus gigas* NC_006891.1, *Pyrhila pisum* NC_030047.1, *Scylla olivacea* NC_012569.1, *Scylla paramamosain* NC_012572.1, *Scylla serrata* NC_012565.1, *Scylla tranquebarica* NC_012567.1, *Segonzacia mesatlantica*NC_035300.1, *Sesarma neglectum* NC_031851.1, *Sesarmops sinensis* NC_030196.1, *Sinopotamon xiushuiense* NC_029226.1, *Sinopotamon yaanense* NC_036947.1, *Somanniathelphusa boyangensis* NC_032044.1, *Tubuca arcuata* KX911977.1, *Umalia orientalis* NC_026688.1, *Varuna yui* NC_037155.1, *Xenograpsus ngatama* NC_035951.1, *Xenograpsus testudinatus NC*_013480.1.
